# Diastereoselective Synthesis of Potent Antimalarial *Cis*-β-lactam Agents

**DOI:** 10.22037/ijpr.2017.2024

**Published:** 2019

**Authors:** Aliasghar Jarrahpour, Maryam Rostami, Véronique Sinou, Christine Latour, Lamia Djouhri-Bouktab, Jean Michel Brunel

**Affiliations:** a *Department of Chemistry, College of Sciences, Shiraz University, Shiraz, 71454, Iran.*; b *Aix-Marseille Université, UMR-MD3 Relation hôte-parasites, Physiopathologie & Pharmacologie, Faculté de pharmacie, Bd Jean Moulin, F-13385, Marseille, France.*; c *Centre de Recherche en Cancérologie de Marseille (CRCM), CNRS, UMR7258 ; Institut Paoli Calmettes ; Aix- Marseille Université, UM 105 ; Inserm, U1068, Faculté de pharmacie, Bd Jean Moulin, F-13385, Marseille, France.*

**Keywords:** N-(2-aminoethyl) β-lactams, tert-butyl carbamate, 2-Azetidinones, *P. falciparum*, Staudinger, Antimalarial activity

## Abstract

Fifteen novel β-lactams bearing N-ethyl tert-butyl carbamate group 5a-o and fifteen N-(2- aminoethyl) β-lactams **6a-o **were synthesized by [2+2] ketene-imine cycloaddition reaction (Staudinger). The cycloaddition reaction was found to be totally diastereoselective leading exclusively to theformation of *cis*-β-lactam derivatives. These newly synthesized β-lactams were evaluated for their antimalarial activity against *p. falciparum *K14 resistant strain and showed good to excellent EC50 values. Of the thirty β-lactams tested, **5 h, 6a **and **6c **showed IC50 < 20 µM while **5b**, **5c**, **5e**, **5f**, **5g**, **5i**, **5j**, **6d**, **6g **and **6h **exhibited IC50 <50 . Compounds 5c, 5h, and 5q-t were examined for their anticancer properties against K562 *Leukemia *cell line and 5s showed the best activity. Compounds **3a-j**, **5a-o**, **6a-o**, were tested against *S. aureus ****,***

*E. coli*, *C. albicans *and showed no activity below 125 µg/mL.

## Introduction

Malaria has been a serious public disease for many decades. The development of resistance by *plasmodium falciparum* to drugs such as chloroquine and quinine requires the discovery and development of new drugs (1). A new diterpenoid β-lactam alkaloid showing potent antimalarial activity has been isolated from marine sponge *Hymeniacidonsp* by Rodriguez and his coworker (2). Due to spread of resistance in the mosquito vector to currently available insecticides the control of malaria is becoming more complicated. Therefore, it is necessary to synthesize new classes of antimalarials (3), and to develop them as drugs with varied models of action to overcome the problem of resistance (4). The development of a novel class of antimalarials derived from β-lactam has initiated in recent years (5-6). β-Lactam derivatives with various functional groups have played an important role in antibacterial drugs and in medicinal chemistry (7). The β-lactam ring is an important structural element of the most widely employed β-lactam antibiotics family (8), which includes representative structural classes; penams, cephems, penems, monobactams, carbapenems, and trinems (9). In addition, β-lactams show many important non-antibiotic biological activities (10). They also have increasingly being used as valuable starting materials to develop new synthetic methodologies (11-12). The constant need for potent and effective β-lactam antibiotics as well as more effective β-lactamase inhibitors has prompted synthetic organic and medicinal chemist to design new functionalized 2-azetidinones. There are a large number of synthetic methods for the preparation of β–lactams (13), for which the [2+2] cycloaddition of ketenes with imines (the Staudinger reaction) is the most important method for constructing the 2-azetidinone ring (14).

## Experimental

*General*

All needed chemicals were purchased from Merck, Fluka and Acros. All reagents and solvents were dried prior to use according to standard methods. IR spectra were run on a Shimadzu FT-IR 8300 spectrophotometer. ^1^H-NMR and ^13^C-NMR spectra were recorded in DMSO-d_6_ or CDCl_3_ using a Bruker Avance DPX instrument (^1^H-NMR 250 MHz, ^13^C-NMR 62.9 MHz). Chemical shifts were reported in parts per million (δ) downfield from TMS. All of the coupling constants (J) are in hertz. The mass spectra were recorded on a Shimadzu GC-MS QP 1000 EX instrument. Elemental analyses were run on a Thermo Finnigan Flash EA-1112 series. Melting points were determined in open capillaries with Buchi 510 melting point apparatus. Thin-layer chromatography was carried out on silica gel F254 analytical sheets obtained from Fluka. Column chromatography was performed on Merck Kiesel gel (230–270 mesh).

*General Procedure for Preparation of Schiff Bases* ***3a-j***

A solution of N-tert-butoxycarbonyl-1, 2-ethanediamine (0.50 g, 3.10 mmol) in anhydrous CH_2_Cl_2_ (25 mL) was treated with different aldehydes (3.10 mmol) in the presence of anhydrous MgSO_4_ (6.00 g). The reaction mixture was stirred at room temp for 16 h, filtered and the solvent eliminated under vacuum to give crude Schiff bases 3a-j. They were used for next stage without further purification.

*General procedure for the synthesis of monocyclic β-lactams* ***5a-o***

A solution of acyl chloride (1.2 mmol) in dry CH_2_Cl_2_ (10 mL) was slowly added to a solution of Schiff bases (1.0 mmol) and triethylamine (2 mmol) in CH_2_Cl_2_ (15 mL) at -82 ^o^C. The reaction mixture was then allowed to warm to room temperature, stirred overnight and then the solution was washed successively with HCl 1N (20 mL), saturated NaHCO_3_ (20 mL), and brine (20 mL). Then, it was dried over Na_2_SO_4_ and then filtered. The solvent was evaporated under reduced pressure to give the crude product. All β-Lactams were purified by recrystallization from EtOH, EtOAc except 5j which was purified by column chromatography ethyl acetate /petroleum ether (1:2).

*Tert-butyl2-(2-(4-chlorophenyl)-4-oxo-3-phenoxyazetidin-1-yl)ethylcarbamate (* ***5a*** *)*

White solid (Yield 40%); Mp: 148-150 ^o^C; IR (KBr, cm^-1^): 3385 (NH), 1727 (C=O, β-lactam), 1712 (C=O, BOC), ^1^H-NMR (250 MHz, CDCl_3,_): δ 1.41 (s, 9H), 3.17 (m, 2H), 3.53 (m, 2H,), 5.00 (brs, 1H), 5.08 (d, *J* = 4.4, 1H), 5.38 (d, *J* = 4.4 Hz, 1H), 6.71-7.28 (m, 9H). ^13^C-NMR (62.9 MHz, CDCl_3_) δ = 28.4, 37.9, 41.7, 61.5, 79.6, 81.8, , 115.4, 122.1, 128.5, 129.2, 129.8, 131.6, 134.6, 155.0, 156.7, 166.5. MS (m/z) = 417 [M+H]^+^. Anal. Calcd for C_22_H_25_ClN_2_O_4_: C, 63.38; H, 6.04; N, 6.72. Found: C, 63.50; H, 5.80; N, 7.31.

*Tert-butyl2-(2-(4-nitrophenyl)-4-oxo-3-phenoxyazetidin-1-yl)ethylcarbamate (* ***5b*** *)*

White solid; Mp: 94-98 ^o^C. IR (KBr, cm^-1^): 3359 (NH), 1749 (C=O, β-lactam), 1696 (C=O, BOC). ^1^H-NMR (250 MHz, CDCl_3,_): δ = 1.46 (s, 9H), 2.95 (m, 2H), 3.61 (m, 2H,), 4.96 (brs, 1H), 5.26 (d, *J* = 4.3, 1H), 5.45 (d, *J* = 4.3 Hz, 1H), 6.69-7.17 (m, 5H), 7.5 (d, *J* = 8.7, 2H), 8.14 (d, *J* = 8.7, 2H) ^13^C-NMR (62.9 MHz, CDCl_3_) δ = 28.1, 37.5, 40.6, 60.2, 77.8, 81.7, 114.6, 121.1, 122.9, 129.3, 129.6, 142.0, 147.2, 156.1, 155.5, 165.0. MS (m/z) = 427 [M]^+^. Anal. Calcd for C_22_H_25_N_3_O_6_: C, 61.82; H, 5.90; N, 9.83. Found: C, 57.8; H, 6.31; N, 9.04.

*Tert-butyl 2-(2-oxo-3-phenoxy-4-((E) styryl)azetidin-1-yl)ethylcarbamate (* ***5c*** *)*

White solid; Mp: 138-140 ^o^C. IR (KBr, cm^-1^): 3291 (NH) 1749 (C=O β-lactam) 1696 (C=O, BOC). ^1^H-NMR (250 MHz, CDCl_3,_): δ = 1.40 (s, 9H), 3.17 (m, 2H), 3.47 (m, 2H,), 4.66 (dd, *J* = 4.4, 8.7 Hz 1H), 5.28 (brs, 1H), 5.31 (d, *J* = 4.4 Hz, 1H), 6.19 (dd, *J* = 8.7, 15.8 Hz 1H), 6.95 (d, *J* = 15.8 Hz, 1H) 7.23-7.32 (m, 9H). ^13^C-NMR (62.9 MHz, CDCl_3_) δ = 28.4, 38.3, 41.5, 61.1, 79.4, 81.8, 115.0, 122.0, 122.39, 126.0, 128.39, 128.6, 129.4, 135.8, 137.3, 156.0, 157.0, 166.0. MS (m/z) = 408 [M]^+^.

*Ttert-butyl2-(2-oxo-3-phenoxy-4-p-tolylazetidin-1-yl)ethylcarbamate (* ***5d*** *)*

White solid; Mp: 94-98 ^o^C. IR (KBr, cm^-1^): 3368 (NH) 1727 (C=O β-lactam) 1713 (C=O, BOC).^1^H-NMR (250 MHz, CDCl_3,_): δ = 1.43 (s, 9H), 2.24 (s, 3H) 2.95 (m, 2H), 3.42 (m, 2H,), 5.05 (2H, H-4, NH, this two peaks were overlapped), 5.35 (d, *J* = 3.0, 1H), 6.69-7.22 (m, 9H). ^13^C-NMR (62.9 MHz, CDCl_3_) δ = 28.4, 37.9, 41.7, 61.5, 79.6, 81.8, 115.4, 122.1, 128.5, 129.2, 129.8, 131.6, 134.6, 155.0, 156.7, 166.5. MS (m/z) = 417 [M+H]^+^. Anal. Calcd for C_23_H_28_N_2_O_4_: C, 69.67; H, 7.12; N, 7.07; Found: C, 66.01; H, 6.41; N, 7.5.

*Tert-butyl2-(2-(3-methoxyphenyl)-4-oxo-3-phenoxyazetidin-1-yl)ethylcarbamate (* ***5e*** *)*

White solid; Mp: 110-114 ^o^C. IR (KBr, cm^-1^): 3367 (NH) 1744 (C=O β-lactam) 1716 (C=O BOC). ^1^H-NMR (250 MHz, CDCl_3,_): δ = 1.44 (s, 9H), 3.02 (m, 2H), 3.52 (m, 2H,), 3.74 (s, 3H), 5.05 (2H, H-4, NH this two peaks were overlapped), 5.39 (d, *J* = 4.2, 1H), 6.72-7.25 (m, 9H). ^13^C-NMR (62.9 MHz, CDCl_3_) δ = 28.38, 38.1, 41.8, 55.2, 62.1, 79.6, 81.9, 113.9, 114.4, 115.5, 120.9, 121.9, 129.1, 129.3, 134.6, 156.1, 156.9, 159.5, 166.6. MS (m/z) = 412 [M]^+^.

*Tert-butyl2-(2-oxo-3-phenoxy-4-phenylazetidin-1-yl)ethylcarbomate (* ***5f*** *)*

White solid; Mp: 138-140 ^o^C. IR (KBr, cm^-1^): 3413 (NH) 1756 (C=O β-lactam) 1754 (C=O BOC). ^1^H-NMR (250 MHz, CDCl_3,_): δ = 1.34 (s, 9H), 2.91 (m, 2H), 3.55 (m, 2H,), 5.08 (2H, H-4, NH this two peaks were overlapped), 5.46 (d, *J* = 4.4, 1H), 6.69-7.59 (m, 10H). ^13^C-NMR (62.9 MHz, CDCl_3_) δ = 28.4, 38.1, 41.7, 62.2, 79.6, 81.9, 115.5, 121.9, 128.3, 128.6, 128.7, 129.2, 132.9, 156.3, 156.6, 166.5. MS (m/z) = 382 [M]^+^. Anal. Calcd for C_22_H_26_N_2_O_4_: C, 69.09; H, 6.85; N, 7.32; Found: C, 60.39 H, 5.22; N, 7.78.

*Tert-butyl2-(2-(3-bromophenyl)-4-oxo*-*3-phenoxyazetidin-1-yl)ethylcarbamate (****5g****)*

White solid; Mp: 148-150 °C. IR (KBr, cm^-1^): 3423 (NH) 1740 (C=O, β-lactam) 1693 (C=O, BOC). ^1^H-NMR (250 MHz, CDCl_3,_): δ = 1.45 (s, 9H), 2.97 (m, 2H), 3.47 (m, 2H,), 4.90 (brs, 1H), 5.06 (d, *J* = 4.3, 1H), 5.39 (d, *J* = 4.3 Hz, 1H), 6.72-7.39 (m, 9H). ^13^C-NMR (62.9 MHz, CDCl_3_) δ = 28.4, 37.5, 41.8, 61.4, 79.7, 81.7, 115.4, 122.1, 122.4, 127.2, 129.3, 129.8, 131.5, 131.8, 135.5, 155.8, 156.0, 166.5. MS (m/z) = 462 [M+H]^+^.

*Tert-butyl2-(2-(naphthalen-2-yl)-4-oxo-3-phenoxyazetidin-1-yl)ethylcarbamate(* ***5h*** *)*

White solid; Mp: 134-136 ^o^C. IR (KBr, cm^-1^): 3451 (NH) 1748 (C=O, β-lactam) 1707 (C=O, BOC). ^1^H-NMR (250 MHz, CDCl_3,_): δ = 1.43 (s, 9H), 3.04 (m, 2H), 3.54 (m, 2H,), 4.96 (brs, 1H), 5.25 (d, *J* = 4.3, 1H), 5.46 (d, *J* = 4.3 Hz, 1H), 6.72-7.71 (m, 12H). ^13^C-NMR (62.9 MHz, CDCl_3_) δ = 28.4, 38.1, 41.8, 62.4, 79.5, 82.1, 115.5, 122.9, 125.6, 126.3, 126.4, 127.7, 127.9, 128.1, 128.4, 129.2, 130.7, 133.0, 133.4, 156.2, 156.9, 166.9. Anal. Calcd for C_26_H_28_N_2_O_4_: C, 69.09; H, 6.85; N, 7.32; Found: C, 71.24 H, 6.11; N, 7.06

*Tert-butyl2-(2-(2,3-dimethoxyphenyl)-4-oxo-3-phenoxyazetidin-1-yl)ethylcarbamate (* ***5i*** *)*

White solid; Mp: 110-112 ^o^C. IR (KBr, cm^-1^): 3335 (NH) 1715 (C=O, β-lactam) 1691 (C=O, BOC). ^1^H-NMR (250 MHz, CDCl_3,_): δ = 1.46 (s, 9H), 3.06 (m, 2H), 3.47 (m, 2H,), 3.53-3.79 (s, 6H), 5.25 (brs, 1H), 5.39 (d, *J* = 4.3, 1H), 5.48 (d, *J* = 4.3 Hz, 1H), 6.79-7.18 (m, 5H), 7.5 (d, *J* = 8.7, 2H), 8.14 (d, *J* = 8.7, 2H) ^13^C-NMR (62.9 MHz, CDCl_3_) δ = 28.4, 38.3, 42.1, 55.7, 56.3, 61.0, 79.5, 82.1, 112.6, 115.7, 120.2, 121.9, 123.7, 126.8, 129.2, 148.1, 152.4, 156.0, 157.1, 166.9. MS (m/z) = 442 [M]^+^. Anal. Calcd for C_24_H_30_N_2_O_6_: C, 65.14; H, 6.83; N, 6.33; Found: C, 65.53 H, 7.01; N, 7.07

*Tert-butyl2-(2-(3-nitrophenyl)-4-oxo-3-phenoxyazetidin-1-yl)ethylcarbamate (* ***5j*** *)*

Light brown oil; IR (KBr, cm^-1^): 3358 (NH) 1763 (C=O, β-lactam) 1698 (C=O, BOC). ^1^H-NMR (250 MHz, CDCl_3,_): δ = 1.42 (s, 9H), 2.96 (m, 2H), 3.53 (m, 2H,), 5.08 (brs, 1H), 5.27 (d, *J* = 4.3, 1H), 5.45 (d, *J* = 4.3 Hz, 1H), 6.68-8.20 (m, 9H). ^13^C-NMR (62.9 MHz, CDCl_3_) δ = 41.9, 43.9, 61.6, 79.6, 81.8, 115.1, 121.9, 122.2, 123.5, 123.6, 129.3, 134.4, 135.8, 148.0, 156.3, 156.4, 166.3. MS (m/z) = 427 [M]^+^.

*Tert-butyl 2-(2-(4-chlorophenyl)-3-methoxy-4-oxoazetidin-1-yl)ethylcarbamate (* ***5k*** *)*

White solid; Mp: 100-104 ^o^C. IR (KBr, cm^-1^): 3309 (NH) 1748 (C=O, β-lactam) 1692 (C=O, BOC). ^1^H-NMR (250 MHz, CDCl_3,_): δ = 1.43 (s, 9H), 2.91 (m, 2H) 3.13 (s, 3H), 3.43 (m, 2H,), 4.63 (d, *J* = 4.2, 1H), 4.84 (d, *J* = 4.2, 1H), 5.00 (brs, 1H), 7.26-7.45 (m, 4H). ^13^C-NMR (62.9 MHz, CDCl_3_) δ = 28.4, 37.9, 41.4, 58.2, 61.1, 79.4, 85.6, 129.7, 130.6, 132.3, 134.5, 156.1, 167.6. Anal. Calcd for C_17_H_23_ClN_2_O_4_: C, 57.54; H, 6.53; Cl, 9.99; N, 7.89; Found: C, 58.32 H, 6.74; N, 8.65.

*Tert-butyl 2-(3-methoxy-2-(naphthalen-2-yl)-4-oxoazetidin-1-yl)ethylcarbamate (* ***5l*** *)*

White solid; Mp: 108-110 ^o^C. IR (KBr, cm^-1^): 3355 (NH) 1761 (C=O, β-lactam) 1705 (C=O, BOC). ^1^H-NMR (250 MHz, CDCl_3,_): δ = 1.43 (s, 9H), 2.99 (m, 2H), 3.15 (s, 3H), 3.51 (m, 2H,), 4.74 (d, *J* = 4.3, 1H), 4.95 (brs, 1H), 5.02 (d, *J* = 4.3 Hz, 1H), 7.46-7.89 (m, 7H). ^13^C-NMR (62.9 MHz, CDCl_3_) δ = 28.3, 38.2, 41.5, 58.2, 62.1, 79.5, 85.8, 125.5, 126.4, 127.7, 127.9, 128.1, 128.2, 131.3, 133.1, 133.5, 156.1, 167.6. MS (m/z) = 369 [M+H]^+^.

*Tert-butyl 2-(3-methoxy-2-(4-nitrophenyl)-4-oxoazetidin-1-yl)ethylcarbamate (* ***5m*** *)*

White solid; Mp: 112-114 ^o^C. IR (KBr, cm^-1^): 3340 (NH) 1754 (C=O, β-lactam) 1683 (C=O, BOC). ^1^H-NMR (250 MHz, CDCl_3,_): δ = 1.40 (s, 9H), 2.89 (m, 2H), 3.15 (s, 3H), 3.41 (m, 2H,), 4.69 (d, *J* = 4.4, 1H), 5.03 (d, *J* = 4.4, 1H), 5.28 (brs, 1H), 7.58 (d, *J* = 8.8 Hz, 2H), 8.21 (d, *J* = 8.8 Hz, 2H).^13^C-NMR (62.9 MHz, CDCl_3_) δ = 28.3, 37.8, 41.5, 58.4, 60.1, 79.4, 86.0, 123.5, 129.2, 141.8, 147.9, 156.2, 167.4. Anal. Calcd for C_17_H_23_N_3_O_6_: C, 55.88; H, 6.34; N, 11.50; Found: C, 56.48 H, 6.63; N, 12.36.

*Tert-butyl2-(3-methoxy-2-(3-methoxyphenyl)-4-oxoazetidin-1-yl)ethylcarbamate (* ***5n*** *)*

White solid; Mp: 111-113 ^o^C. IR (KBr, cm^-1^): 3348 (NH) 1764 (C=O, β-lactam) 1699 (C=O, BOC). ^1^H-NMR (250 MHz, CDCl_3,_): δ = 1.42 (s, 9H), 2.96 (m, 2H), 3.13 (s, 3H), 3.39 (m, 2H,), 3.77 (s, 3H), 4.63 (d, *J* = 4.3, 1H), 4.83 (d, *J* = 4.3, 1H), 5.03 (brs, 1H), 6.86-7.32 (m, 4H). ^13^C-NMR (62.9 MHz, CDCl_3_) δ = 28.3, 37.9, 41.4, 55.1, 58.1, 61.7, 79.2, 85.6, 113.7, 113.9, 120.6, 129.4, 135.4, 159.6, 156.1, 167.6. Anal. Calcd for C_18_H_26_N_2_O_5_: C, 61.70; H, 7.48; N, 7.99; Found: C, 62.78 H, 7.65; N, 8.98.

*Tert-butyl2-(3-methoxy-2-oxo-4-phenylazetidin-1-yl)ethylcarbamate (* ***5o*** *)*

White solid; Mp: 110-113 ^o^C. IR (KBr, cm^-1^): 3330 (NH) 1744 (C=O, β-lactam) 1693 (C=O, BOC). ^1^H-NMR (250 MHz, CDCl_3,_): δ = 1.22 (s, 9H), 2.64 (m, 2H), 2.85 (s, 3H), 3.17 (m, 2H,), 4.36 (d, *J* = 4.0, 1H), 4.59 (d, *J* = 4.0, 1H), 4.99 (brs, 1H), 7.01-7.12 (m, 5H). ^13^C-NMR (62.9 MHz, CDCl_3_) δ = 28.3, 38.0, 41.3, 58.0, 61.8, 79.2, 85.6, 128.3, 128.4, 128.6, 133.7, 156.1, 167.7. Anal. Calcd for C_17_H_24_N_2_O_4_: C, 63.73; H, 7.55; N, 8.74; Found: C, 56.71 H, 7.07; N, 8.64

*General procedure for the deprotection of BOC protecting group*

A solution of β-lactams (***5a-o***) (0.5 mmol) in CH_2_Cl_2_ (12 mL) was cooled to 0 °C and treated with TFA (3-5 mmol). After the addition, the cooling bath was removed and stirring was continued until total disappearance of the starting material (TLC). Then the solution was basified with 5% aqueous NaOH solution (pH = 10). The organic layer was separated, and the aqueous layer was extracted with CH_2_Cl_2_ (2 × 30 mL). The combined extracts were washed with brine, dried over Na_2_SO_4_, and concentrated to give crude N-(2-aminoethyl) β-lactams 6a-o. 

*1-(2-Aminoethyl)-4-(4-chlorophenyl)-3-phenoxyazetidin-2-one (* ***6a*** *)*

White solid; IR (KBr, cm^-1^): 3349, 3417 (NH_2_) 1734 (C=O, β-lactam). ^1^H-NMR (250 MHz, DMSO): δ = 2.50-3.03 (m, 4H), 2.47 (s, 2H), 5.18 (d, *J* = 4.4, 1H), 5.60 (d, *J* = 4.4, 1H), 6.71-7.17 (m, 9H). ^13^C-NMR (62.9 MHz, DMSO) δ = 45.0, 49.1, 65.7, 86.3, 120.1, 126.8, 133.2, 134.4, 135.4, 137.9, 138.4, 161.5, 170.4.

*1-(2-Aminoethyl)-4-(4-nitrophenyl)-3-phenoxyazetidin-2-one (* ***6b*** *)*

White solid; IR (KBr, cm^-1^): 3350, 3301 (NH_2_) 1739 (C=O, β-lactam). ^1^H-NMR (250 MHz, DMSO): δ = 2.59 (s, 2H), 2.88-3.03 (m, 4H), 5.30 (d, *J* = 4.3, 1H), 5.65 (d, *J* = 4.3, 1H), 6.88-8.17 (m, 5H), 6.73 (d, *J* = 8.6, 2H),7.59 (d, *J* = 8.6, 2H). ^13^C-NMR (62.9 MHz, DMSO) δ = 48.6, 53.6, 65.7, 86.7, 120.1, 126.7, 133.7, 134.5, 134.7, 134.8, 147.5, 161.3, 176.9.

*1-(2-Aminoethyl)-3-phenoxy-4-styrylazetidin-2-one (* ***6c*** *)*

White solid; IR (KBr, cm^-1^): 3436 , 3378 (NH_2_) 1744 (C=O, β-lactam).^1^H-NMR (250 MHz, DMSO): δ = 2.68-3.09 (m, 4H), 2.40 (s, 2H), 4.67 (dd, *J* = 4.4, 8.8, 1H), 5.46 (d, *J* = 4.4, 1H), 6.13 (dd, *J* = 8.8, 15.9, 1H), 6.89 (d, *J* = 15.9, 1H ), 7.21-7.86 (m, 10H). ^13^C-NMR (62.9 MHz, DMSO) δ = 40.1, 44.0, 61.6, 81.9, 128.4, 115.6, 122.1, 122.5, 126.7, 128.4, 129.4, 135.8, 137.3, 157.0, 166.1.

*1-(2-Aminoethyl)-3-phenoxy-4-p-tolylazetidin-2-one (* ***6d*** *)*

White solid; IR (KBr, cm^-1^): 3344, 3280 (NH_2_) 1745 (C=O, β-lactam). ^1^H-NMR (250 MHz, DMSO): δ = 2.23 (s, 3H) 2.47 (s, 2H), 2.80-3.35 (m, 4H), 5.11 (d, *J* = 4.40 1H), 5.56 (d, *J* = 4.0, 1H), 6.72-7.92 (m, 9H). ^13^C-NMR (62.9 MHz, DMSO) δ = 20.6, 40.2, 43.5, 61.06, 81.1, 115.2, 121.5, 126.8, 128.2, 128.6, 130.9, 137.3, 165.4.

*1-(2-Aminoethyl)-4-(3-methoxyphenyl)-3-phenoxyazetidin-2-one (* ***6e*** *)*

White solid; IR (KBr, cm^-1^): 3367, 3302 (NH_2_) 1753 (C=O, β-lactam). ^1^H-NMR (250 MHz, DMSO): δ = 2.47 (s, 2H), 2.58-3.47 (m, 4H), 3.64 (s, 3H), 5.12 (d, *J* = 4.4, 1H), 5.59 (d, *J* = 4.4, 1H), 6.73-7.24 (m, 9H). ^13^C-NMR (62.9 MHz, DMSO) δ = 43.0, 44.3, 60.2, 61.7, 81.7, 113.3, 114.4, 115.7, 120.9, 122.0, 129.5, 129.7, 156.1, 159.3, 165.8, 166.6.

*1-(2-Aminoethyl)-3-phenoxy-4-phenylazetidin-2-on (* ***6f*** *)*

White solid; IR (KBr, cm^-1^): 3330, 3290 (NH_2_) 1749 (C=O, β-lactam). ^1^H-NMR (250 MHz, DMSO): δ = 2.51 (s, 2H), 2.47-3.39 (m, 4H), , 5.16 (d, *J* = 4.3, 1H), 5.60 (d, *J* = 4.3, 1H), 6.73-7.31 (m, 10H). ^13^C-NMR (62.9 MHz, DMSO) δ = 40.1, 43.1, 61.2, 80.9, 115.2, 122.9, 128.1, 128.2, 129.2, 129.5, 133.7, 156.3, 165.7.

*1-(2-Aminoethyl)-4-(3-bromophenyl)-3-phenoxyazetidin-2-one (* ***6g*** *)*

White solid; IR (KBr, cm^-1^): 3348, 3293 (NH_2_) 1742 (C=O, β-lactam). ^1^H-NMR (250 MHz, DMSO): δ = 2.46 (s, 2H), 2.60-3.54 (m, 4H), 5.18 (d, *J* = 4.4, 1H), 5.62 (d, *J* = 4.4, 1H), 6.72-7.94 (m, 9H). ^13^C-NMR (62.9 MHz, DMSO) δ = 40.0, 42.5, 60.2, 81.4, 115.3, 121.6, 127.3, 129.2, 129.7, 130.0, 130.1, 130.9, 136.9, 157.2, 171.9.

*1-(2-aminoethyl)-4-(naphthalen-2-yl)-3-phenoxyazetidin-2-one (* ***6h*** *)*

White solid; IR (KBr, cm^-1^): 3359, 3290 (NH_2_) 1739. (C=O, β-lactam). ^1^H-NMR (250 MHz, DMSO): δ = 2.47 (s, 2H), 2.58-3.48 (m, 4H), 5.33 (d, *J* = 4.4, 1H), 5.67 (d, *J* = 4.4, 1H), 6.72-7.86 (m, 9H). ^13^C-NMR (62.9 MHz, DMSO) δ = 40.3, 43.4, 61.2, 81.3, 115.3, 121.5, 125.4, 125.7, 126.0, 127.4, 129.2, 129.5, 131.7, 132.6, 139.9, 156.4, 165.5.

*1-(2-aminoethyl)-4-(2,3-dimethoxyphenyl)-3-phenoxyazetidin-2-one (* ***6i*** *)*

White solid; IR (KBr, cm^-1^): 1751 (C=O, β-lactam). ^1^H-NMR (250 MHz, DMSO): δ = 1.90 (s, 2H), 2.60-3.65 (m, 4H), 3.68-3.75 (s, 6H), 5.43 (d, *J* = 4.6, 1H), 5.62 (d, *J* = 4.6, 1H), 6.73-7.19 (m, 8H). ^13^C-NMR (62.9 MHz, DMSO) δ = 42.0, 49.8, 53.1, 55.4, 60.2, 81.1, 112.6, 115.1, 119.6, 121.8, 126.7, 129.3, 136.1, 151.9, 156.6, 157.4, 166.3.

*1-(2-Aminoethyl)-4-(3-nitrophenyl)-3-phenoxyazetidin-2-one (* ***6j***)

White solid; IR (KBr, cm^-1^): 3516, 3358 (NH_2_) 1749 (C=O, β-lactam). ^1^H-NMR (250 MHz, DMSO): δ = 2.6 (s, 2H), 2.89-3.41 (m, 4H), 5.39 (d, *J* = 4.3, 1H), 5.71

 (d, *J* = 4.3, 1H), 6.71-7.80 (m, 8H). ^13^C-NMR (62.9 MHz, DMSO) δ = 42.6, 48.2, 60.2, 83.3, 115.3, 121.4, 121.7, 121.9, 122.1, 129.4, 134.1, 144.0, 147.5, 157.1, 171.6.

*1-(2-Aminoethyl)-4-(4-chlorophenyl)-3-methoxyazetidin-2-one (* ***6k*** *)*

White solid; IR (KBr, cm^-1^): 3497, 3358, (NH_2_) 1743 (C=O, β-lactam). ^1^H-NMR (250 MHz, DMSO): δ = 2.45 (s, 2H), 3.0 (s, 3H), 2.49-2.91 (m, 4H), 4.68 (d, *J* = 4.4, 1H), 4.87 (d, *J* = 4.4, 1H), 7.28-7.65 (m, 4H).^ 13^C-NMR (62.9 MHz, DMSO) δ = 40.0, 57.2, 60.1, 85.1, 129.2, 131.2, 132.7, 133.9, 166.6.

*1-(2-Aminoethyl)-3-methoxy-4-(naphthalen-2-yl)azetidin-2-one (* ***6l*** *)*

White solid; IR (KBr, cm^-1^): 3504, 3320 (NH_2_) 1740 (C=O, β-lactam). ^1^H-NMR (250 MHz, DMSO): δ = 2.46 (s, 2H), 2.9 (s, 3H), 2.47-3.48 (m, 4H), 4.81 (d, *J* = 4.4, 1H), 5.04 (d, *J* = 4.4, 1H), 7.43-7.91 (m, 7H). ^13^C-NMR (62.9 MHz, DMSO) δ = 40.1, 42.8, 57.3, 61.1, 85.4, 125.2, 125.7, 126.1, 126.2, 127.2, 127.5, 127.6, 127.7, 132.6, 132.7, 166.7.

*1-(2-Aminoethyl)-3-methoxy-4-(4-nitrophenyl)azetidin-2-one (* ***6m*** *)*

White solid; IR (KBr, cm^-1^): 3506, 3358 (NH_2_) 1742 (C=O, β-lactam). ^1^H-NMR (250 MHz, DMSO): δ = 2.48 (s, 2H), 2.88 (s, 3H), 2.47-2.96 (m, 4H), 4.83 (d, *J* = 4.4, 1H), 5.07 (d, *J* = 4.4, 1H), 7.67 (d, *J* = 8.8, 2H), 8.13 (d, *J* = 8.8, 2H). ^13^C-NMR (62.9 MHz, DMSO) δ = 42.6, 48.4, 59.1, 60.4, 87.6, 123.1, 128.4, 146.2, 147.2, 167.4.

*1-(2-Aminoethyl)-3-methoxy-4-(3-methoxyphenyl)azetidin-2-one (* ***6n*** *)*

White solid; IR (KBr, cm^-1^): 3514, 3330 (NH_2_) 1755 (C=O, β-lactam). ^1^H-NMR (250 MHz, DMSO): δ = 3.02 (s, 2H), 3.25 (s, 3H), 2.56-3.31 (m, 4H), 3.71 (s, 3H), 4.70 (d, *J* = 4.3, 1H), 4.83 (d, *J* = 4.3, 1H), 6.83-7.28 (m, 4H). ^13^C-NMR (62.9 MHz, DMSO) δ = 28.3, 37.9, 41.4, 55.1, 58.1, 61.7, 85.6, 113.7, 113.9, 120.6, 129.4, 135.4, 159.6, 166.7.

*1-(2-Aminoethyl)-3-methoxy-4-phenylazetidin-2-one (* ***6o***)

White solid; IR (KBr, cm^-1^): 3497, 3358 (NH_2_) 1743 (C=O, β-lactam). ^1^H-NMR (250 MHz, DMSO): δ = 2.47 (s, 2H), 2.99 (s, 3H), 2.53-3.79 (m, 4H), 4.78 (d, *J* = 4.4, 1H), 4.94 (d, *J* = 4.4, 1H), 7.30-7.45 (m, 5H). ^13^C-NMR (62.9 MHz, DMSO) δ = 42.6, 49.2, 57.1, 60.5, 88.8, 127.8, 128.0, 134.7, 142.9, 166.7.

*General procedure for antimalarial activity measurements*

The chloroquine-resistant *P. falciparum *strain K14 (Southeast Asia) was cultured in vitro in complete medium consisting of RPMI 1640 (In Vitrogen) supplemented with 27.5 mM NaHCO_3_, 20 mg/L gentamycin, and 10% human serum (19). Parasites were grown at 37 °C in human O+ red blood cells at a 6% hematocrit under a 5% CO2, 10% O2 and 85% N2 atmosphere. Cultures were synchronized by sorbitol treatments (20). Stock solutions of lactam derivatives were prepared in sterile DMSO (10 mM) and later dilutions were with complete culture medium. Increasing concentrations of lactam derivatives (100 µL/well, top concentration = 50 µM) were distributed in a 96-well plate; DMSO (0.5% vol/vol, top concentration) was distributed for control. Then, 100 µL from a culture containing > 95% ring (0-20 h postinvasion) at a 0.8% parasitemia and 3% haematocrit in complete medium was added per well along with 1.0 µCi of 3H-hypoxanthine with a specific activity of 14.1 Ci/mmol (Perkin-Elmer, Courtaboeuf, France). Parasites were grown for 42 h at 37 °C. Plates were then freeze-thawed and harvested on filters. Dried filters were moistened in scintillation liquid mixture (Microscint O; Perkin-Elmer) and counted in a Top Count Microbeta counter (Perkin-Elmer). Percentage growth inhibition was calculated from the parasite-associated radioactivity. 100% 3H-hypoxanthine incorporation was determined from a control grown in the absence of lactam derivatives. The concentration of drug giving 50% inhibition of label incorporation (IC50) was determined by nonlinear regression analysis of log-based dose-response curve (Riasmart; Packard). Each concentration was estimated from independent experiments in triplicate.

**Scheme 1:**
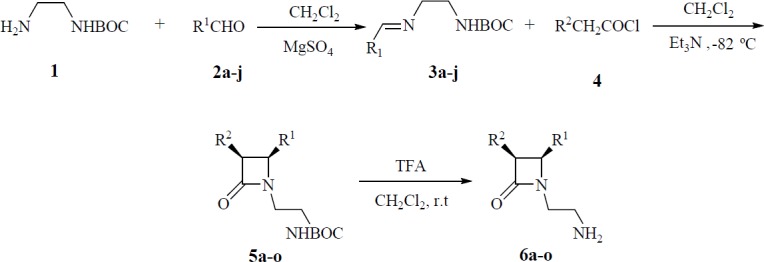
Synthesis of *cis*-β-lactams **5a-o **and **6a-o**

**Table 1:** Isolated yields for *cis*-β-lactams **5a-o **and **6a-o**

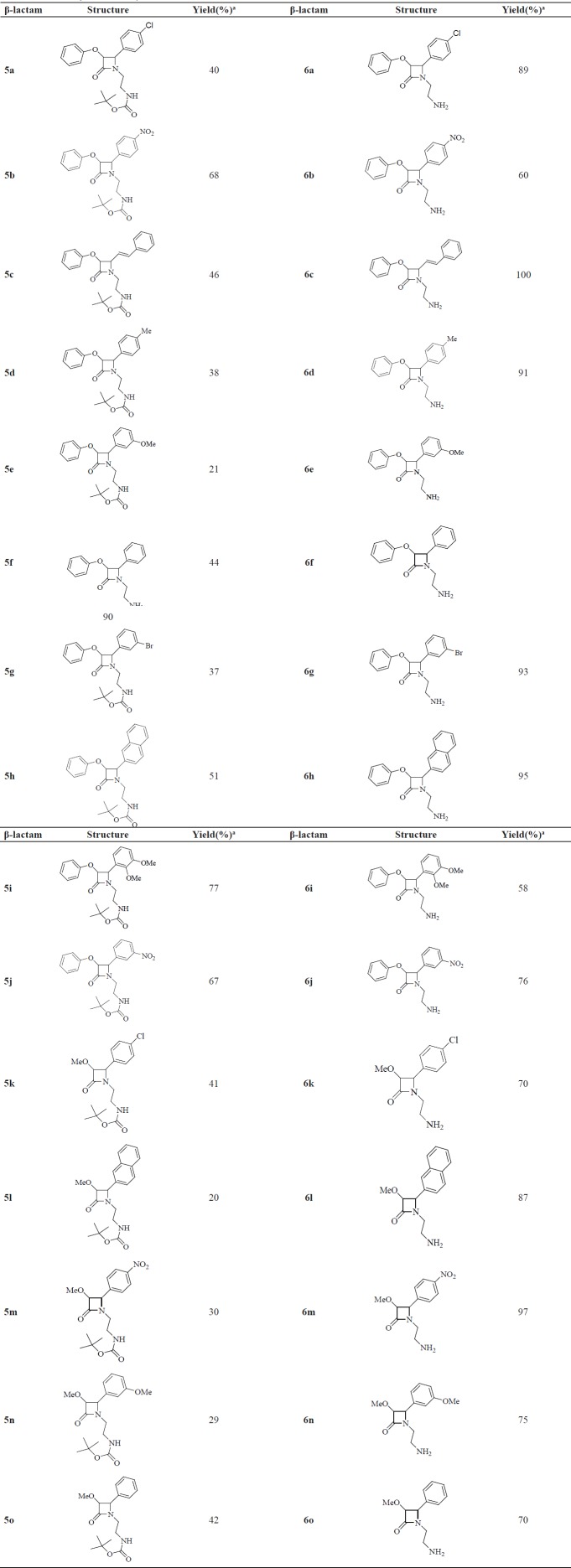

**Table 2:** Antimalarial activity of the new *cis*-2-azetidinones **5a-o **and β-lactams **6a-o**.

**Compound**	**IC** **50 ** **(µM) ** ***P. falciparum K14***	**Compound**	**IC** **50 ** **(µM) ** ***P. falciparum K14***
Chloroquine	11	**6a**	19
**5a**	>50	**6b**	>50
**5b**	27	**6c**	15
**5c**	24	**6d**	22
**5d**	>50	**6e**	>50
**5e**	30	**6f**	>50
**5f**	32	**6g**	37
**5g**	35	**6h**	21
**5h**	16	**6i**	>50
**5i**	21	**6j**	>50
**5j**	23	**6k**	>50
**5k**	>50	**6l**	>50
**5l**	>50	**6m**	>50
**5m**	>50	**6n**	>50
**5n**	>50	**6o**	>50
**5o**	>50		

## Results and discussion

A mixture of amine 1 which was prepared from ethylenediamine and di-tert-butyl dicarbonate ((BOC)_2_O) (15), and substituted benzaldehydes (2a-j) in anhydrous dichloromethane (DCM) and MgSO_4_ was stirred at room temperature to give the crude imines 3a-j (Scheme 1). N-BOC protected β-lactams 5a-o were then synthesized by the Staudinger reaction. For this, to a mixture of imines 3a-j and triethylamine, substituted acetyl chlorides 4 were added dropwise at -82 ^º^C to afford crude β-lactams 5a-o. These β-lactams were purified by recrystallization from either ethanol or ethyl acetate in moderate to good yields (Table 1). 

The cycloadducts were characterized by spectral analysis. For 5b, the IR spectrum showed the characteristic absorption of a β-lactam carbonyl at 1749 cm^−1^ and the carbonyl signal of carbamate at 1696 cm^−1^ as well as the NH signal at 3359 cm^−1^. The ^1^H-NMR spectrum exhibited the t-butoxy protons at 1.46 ppm, the β-lactam ring H-3 and H-4 protons resonated as two doublets at 5.45 (*j* = 4.3) and 5.26 (*j* = 4.3) respectively. The *cis* and *trans* stereochemistries of 2-azetidinones were deduced from coupling constants of H-3 and H-4 (*J*_3,4_>4.0 Hz for the cis and *J*_3,4_<3.0 Hz for the *trans* stereoisomer) (16-17). The NH signal was exhibited at 4.96 ppm as a broad peak in CDCl_3_ eliminated when 5b was vigorously shaken with D_2_O. The ^13^C-NMR spectrum of 5b exhibited the C-3 and C-4 of β-lactam ring at 81.7, 60.2 respectively, C=O (β-lactam) at 165.0 and (C=O, BOC) at 155.5. Subsequent treatment of β-lactams 5a-o with trifluoroacetic acid (TFA) in dry CH_2_Cl_2_ at room temperature afforded the deprotected N-(2-aminoethyl) β-lactams 6a-o (Table1).

Removal of the BOC residue resulted in removing of t-butoxy signal in ^1^H-NMR and the appearance of the NH_2_ peaks in IR spectra. The removal of the BOC was also confirmed by mass spectra and elemental analyses. Thus, for example the ^1^H-NMR spectrum of 6a showed β-lactam H-3 and H-4 protons as two doublets at 5.60 ppm (*J* = 4.4 Hz) and 5.18 ppm (*J* = 4.4 Hz), respectively confirming the *cis* stereochemistry for these free amino β-lactam. The IR spectrum of this compound displayed NH_2_ peaks at 3349, and 3417 and β-lactam carbonyl at 1734 cm ^-1^. The X-ray crystal structure of a *para*-methoxyphenyl derivative which is very close to 5d and 6d is shown in Figure 1. 

The central β-lactam ring is almost planar. The methoxyphenyl ring is almost coplanar with β-lactam ring, whereas the tolyl ring is almost normal to it. The dihedral angle between β-lactam ring and O-bonded phenyl ring is 51.95 (18).

*Biological activities*

*Antibacterial and antifungal activities*

Compounds 3a-j, **5a-o**, **6a-o**, were tested against *S. aureus* ***, ****E. coli*, and *C. albicans* showing no activity below 125 µg/mL.In another assay, compounds **5a-i** were tested against four American Type Culture Collection (ATCC) strains *C. albicans* (ATCC10261), *A. flavus* (ATCC64025), *S. aureus* (ATCC 25923) ,and *E. coli* (ATCC 25922) showing no activity below 256 µg/mL 

*Anticancer activity*

Compounds **5c**, **5h** and **5q-t**, were examined for their anticancer properties against K562 *Leukemia* cell line at two different concentrations (50 and 100 µg/mL) and 5s showed the best activity.

*Antimalarial activity*

Good to excellent antimalarial activities have been obtained against chloroquine resistant *p. falciparum* K14 strain as outlined in Table 2 for *cis*-2-azetidinones 5a-o and β-lactams 6a-o with IC_50_ varying from 15 to 50 µM in the better cases. Thus, even if the mechanism of action of these compounds remains unknown some structure-activity relationships can be underlined. Firstly, it is noteworthy that the less active derivatives 5k-5o and 6k-6o differ from the other derivatives by the replacement on the lactam ring of a phenoxy group by a methoxy one suggesting a dramatic contribution of this moiety on the encountered antimalarial activity. On the other hand, a slight change such as removal of the BOC protecting group led to an increase of the biological activity of compounds 6a and 6d (IC_50_ = 19 and 22 µM respectively) whereas their protected parent derivatives are totally inactive suggesting here again a quite strong influence of the structure of the considered lactam derivative on the mechanism of action.

## Conclusion

In this study, thirty novel β-lactams bearing the N-ethyl tert-butyl carbamate and N-(2-aminoethyl) β-lactams were synthesized by [2+2] ketene-imine cycloaddition reaction (Staudinger). The cycloaddition reaction was found to be totally diastereoselective leading exclusively to formation of *cis*-β-lactam derivatives. These newly synthesized β-lactams were evaluated for their antimalarial activity against *p. falciparum* K14 resistant strain and showed good to excellent EC_50_ values. Of the thirty β-lactams tested, **5h**, **6a** and **6c** showed IC_50 _< 20 µM while **5b**, **5c**, **5e**, **5f**, **5g**, **5i**, **5j**, **6d**, **6g** and **6h** exhibited IC_50_ < 50. Compounds **5c**, **5h**, and **5q-t** were examined for their anticancer properties against K562 *Leukemia* cell line and 5s showed the best activity. Compounds **3a-j**, **5a-o**, **6a-o** were tested against *S. aureus* (ATCC 25923), *E. coli *(ATCC 25922), *C. albicans* (ATCC10261) and showed no activity below 125 µg/mL.
